# Platinum-Based Cytostatics Used in Oncology with Respect to Environmental Fate and Innovative Removal Strategies of Their Metabolites

**DOI:** 10.3390/molecules31010168

**Published:** 2026-01-01

**Authors:** Rafał Olchowski, Kinga Morlo, Ryszard Dobrowolski

**Affiliations:** 1 Department of Pharmacology, Toxicology and Environmental Protection, Faculty of Veterinary Medicine, University of Life Sciences, Akademicka Sq. 12, 20-950 Lublin, Poland; rafal.olchowski@up.lublin.pl; 2 Department of Analytical Chemistry, Institute of Chemical Sciences, Faculty of Chemistry, Maria Curie-Sklodowska University, M. C. Sklodowska Sq. 3, 20-031 Lublin, Poland; kinga.morlo@mail.umcs.pl

**Keywords:** cisplatin, carboplatin, oxaliplatin, mechanism of action, removal, adsorption, metabolites, urine, hospital wastewater

## Abstract

Pt complexes have been used in human and veterinary oncology for more than 50 years and represent one of the most significant groups of cytostatics. There are a lot of Pt-based compounds, such as cisplatin, carboplatin and oxaliplatin, which exhibit high efficiency against many tumors. Their broad application in oncology medicine and improper waste disposal induce environmental pollution by platinum cytostatics and their metabolites. They can cause toxic effects to fauna and flora, even at low concentration levels. Currently used technologies for wastewater treatment are not sufficient in the case of platinum-based metabolites. Their high resistance and toxicity of their degradation byproducts pose a serious problem. In this review, currently applied platinum-based cytostatics, their possible metabolic mechanisms, environmental impact and technological methods for their removal from wastewater and patients’ urine are summarized. Special attention is paid to adsorption methods.

## 1. Introduction

Cancer is considered as one of the most widespread lethal diseases in humans and animals. In 2020, almost 20 mln people found out about their cancer diagnosis, and in the same time, about 10 mln people died due to cancer. It is estimated that at least 29 mln new cases/year will be present by 2040 [1]. This problem also pertains to animals, such as dogs and cats. There are plenty of tumors, and potentially every living cell can be transformed into a cancer cell. There are various strategies of cancer treatment: radiotherapy, surgery, chemotherapy and combined techniques. In chemotherapy, anticancer drugs are used, which can be divided to some specific groups: cytotoxic chemotherapeutic agents, molecular targeted therapeutics, immunotherapy drugs, cell and gene therapy drugs, hormonal therapeutics and antibody–drug conjugates. Platinum-based drugs are one of the cytotoxic chemotherapeutic agents, which damage DNA (deoxyribonucleic acid) of cancer cells, inhibit DNA synthesis and disrupt cell division [2]. They are the most employed drugs in cancer treatments [3].

Platinum-based drugs are mainly excreted with the patient’s urine in its unchanged or metabolized form and pass into the hospital and urban wastewater [3]. The annual Pt emission by hospitals is 1.3–14.3 kg, which is up to 12% of the estimated amount of Pt emitted by cars with mounted catalytic converters in Europe [4]. The concentration level of Pt metabolites in wastewater is in trace amounts (even ng/L). They are recalcitrant and highly toxic for all living organisms. Chronic exposure to platinum-based metabolites can lead to serious health problems [3]. Thus, efficient removal technologies should be applied. Nowadays, the wastewater treatment plant technology dedicated for hospital and municipal wastewater is based on the solid particle separation, biological, membrane and advanced oxidation methods [3,5]. Unfortunately, the conventional technology of wastewater treatment is insufficient for platinum-containing cytostatic metabolites. These compounds are found in the wastewater treatment plant effluents, which are directly pumped into the environment. Biological treatment is non-resistant for the cytotoxicity of platinum-based metabolites [3]. Furthermore, oxidation methods only change the Pt speciation (e.g., Pt(II) oxidation to Pt(IV)) but without changing the toxicity of the effluent [6]. Thus, the adsorption process is a potentially promising technology for platinum-based metabolite removal from urine and wastewater, especially carbonaceous materials. The adsorbents possess a high specific surface area, on which a large number of selective sites can be introduced. Materials with sulfur-, nitrogen- and oxygen-containing functionalities are promising for this purpose, due to the high affinity towards Pt. Not only adsorption properties but also environmental and economic aspects should be taken into account. Adsorbents with high affinity for Pt should be easily regenerable, to decrease cost of their usage. Moreover, Pt-loaded adsorbents should be properly utilized to prevent Pt emission to the environment. Carbonaceous materials with loaded Pt could be burned and the precious metal could be easily recovered and reused. Another problem is the optimization of the adsorption removal method for large-scale treatment.

In this review, the characteristics of the most applied platinum-based drugs, cisplatin, carboplatin and oxaliplatin, are presented. Their mechanism of action on cancer cells, problem of drug resistance, their metabolites and environmental fate are explained. Moreover, conventional wastewater treatment technology regarding platinum compound removal is broadly described and compared with advanced treatment methods with special attention to adsorption methods. Also, recent studies concerning the efficient degradation of Pt-based metabolites are presented.

## 2. Platinum Pharmaceuticals in Oncology

Platinum complexes are broadly used for treatment of various tumors present both in humans and animals. The kind of platinum drug that is used depends on the treated tumor, the stage of disease, the patient’s condition and the availability of the drug. Starting from the discovery of cisplatin’s cytostatic properties in the 1960s, plenty of platinum compounds have been synthesized with different inorganic and organic ligands (Table 1). But nowadays, the most significant platinum drugs in human and veterinary oncology therapies are cisplatin, carboplatin and oxaliplatin. These compounds are broadly described in the current paragraph.

Cisplatin (cis-diamminedichloridoplatinum(II)) is the first developed platinum-based anticancer agent, which has been approved worldwide since 1979 [7,11]. Cisplatin is administered to almost 50% of human patients struggling with various types of cancer [11]. Cisplatin’s toxicity towards cancer cells can be substantially lowered by means of resistance. The resistance depends on the cancer type. Those highly resistant to cisplatin are non-small-cell lung cancer and colorectal cancer. This resistance to cisplatin’s action can be caused by lowering the cellular uptake of the platinum complex (decreased influx or increased efflux), drug detoxification by cellular thiols and altering the drug target and repairing DNA. Cisplatin resistance can be intrinsic (from the beginning of the treatment) or acquired (over time) [11].

**Figure 1:**
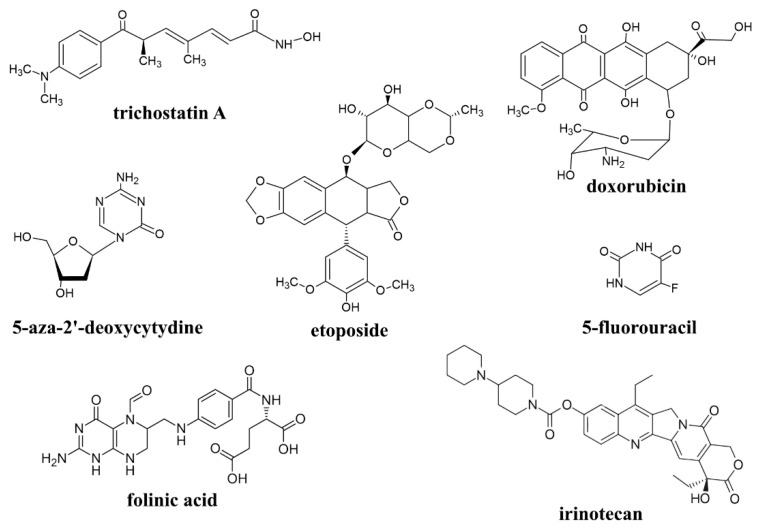
Examples of other anticancer drugs used with platinum-based cytostatics.

Cisplatin is broadly used in antitumor therapy in human oncology. The best effects are observed in the case of small-cell lung cancers. Ovarian cancer is also treated with this platinum complex, but to prevent the cancer from coming back within the next few years after therapy, combination therapy is used. In this kind of therapy, cisplatin is administered to the patient along with honey venom, trichostatin A, 5-aza-2′-deoxycytidine or aferin (Figure 1). High success is also achieved during cisplatin therapy of testicular cancer. For teratoma cancer, the combination of cisplatin with bleomycin and etoposide (Figure 1) is used. But the application of cisplatin is not limited to the treatment of the above-mentioned cancers. It is also used during treatment of childhood brain tumors; gastric, anal, breast, head and neck, prostate, cervical and bladder cancers; leukemia and lymphomas. The doses of cisplatin applied during anticancer therapy are varied from 20 mg/m^2^ to 100 mg/m^2^, which depends on the cancer being treated [11].

In veterinary oncology, cisplatin is also used, mainly in dogs. It is administered via intravenous, intraarterial, intramedullary, intralesional (e.g., with implants) and intracavitary routes. Cisplatin is commonly used as a single agent, though it is occasionally used with doxorubicin (Figure 1) for treatment of osteosarcoma. This platinum drug is also applied during chemotherapy of transitional cell carcinoma and squamous cell carcinoma. Mixed radiotherapy with cisplatin application is studied for treatment of nasosinus carcinomas, nasal tumors and oral melanomas. Cisplatin enhances radiation action, which induces cancer cell apoptosis. The dose of cisplatin administration in dogs is 50–60 mg/m^2^. In horses, therapy with cisplatin is applied for treatment of cutaneous tumors with dosage of 1 mg/cm^3^ of tissue in the target field. Cats are extremely sensitive to cisplatin and acute toxicity induced by this platinum drug is observed. It is probably related to cisplatin-induced dyspnea [12].

Unfortunately, the use of cisplatin in oncology therapy is related to its side effects. The presence of side effects substantially deteriorates the patient’s comfort during oncotherapy. The side effects of cisplatin are revealed in the case of the overdose of this drug (toxic effect). Among the major cisplatin side effects are nephrotoxicity, ototoxicity, hepatotoxicity and gastrointestinal toxicity. Nephrotoxicity induced by cisplatin is related to its renal excretion (90% of total excretion) and drug accumulation in kidneys. The nephrotoxic effect of cisplatin is observed in 28–36% of patients administered with the drug dose of 50 mg/m^2^ [7,11]. Strong hydration is used in the case of dogs treated with cisplatin to minimize the nephrotoxic cisplatin effect [12]. Ototoxicity is observed up to 90% of child patients. In turn, hepatotoxicity is related to cisplatin-induced oxidative stress in hepatocytes. Finally, gastrointestinal toxicity symptoms, like nausea, vomiting, diarrhea, loss of taste or metallic taste and pancreatitis can be present 1–4 h. after cisplatin administration. The use of combination therapy can intensify the gastrointestinal side effects of the cisplatin drug [7,11].

Carboplatin (cis(diammine(1,1-cyclobutanedicarboxylato)platinum(II))) is a second-generation platinum-based cytostatic, which has been applied in oncological therapy since 1989. It has been developed to minimize cisplatin toxicity. It is also intravenously administered in human and veterinary oncology as an aqueous solution diluted in 0.9% NaCl or 5% glucose [7,8,9,10,13]. The resistance mechanisms towards carboplatin are also similar to that of cisplatin. The difference between these two drugs lays in the lower side effects of carboplatin (neurotoxicity and ototoxicity). Thus, the administered dosages of carboplatin can be substantially higher (up to 450 mg/m^2^) than in the case of cisplatin. The maximum dosage of carboplatin is limited by induced myelosuppression and related side effects (leukopenia, neutropenia, thrombocytopenia). In human oncology, carboplatin is the main choice in ovarian cancer treatment. Besides that, it is also used for treatment of other carcinomas, like uterine and non-small-cell lung carcinoma. The combination therapy of carboplatin and other oncologic drugs (e.g., toxoids, vincristine) is used for combating testicular, head and neck, cervical and breast cancers as well as malignant glioma [7,8,9,10]. In turn, during veterinary oncological protocols, carboplatin doses not exceeding 300 mg/m^2^ are used. A wide range of canine malignances (e.g., osteosarcoma, thyroid carcinoma, urogenital and squamous cell cancers) and oral squamous cell carcinoma in cats are treated with carboplatin. Combination therapy using carboplatin is used for overcoming the drug resistance. Similarly to cisplatin, carboplatin is mainly excreted in urine [14,15,16,17,18,19,20].

Oxaliplatin ([(1R,2R)-cyclohexane-1,2-diammine-N,N′](ethanedioato-O,O′)platinum(II)) is a member of the third-generation platinum cytostatics allowed for clinical use in 2002. It is administered intravenously in 5% glucose solution. The applied doses of oxaliplatin lie in the range of 85–130 mg/m^2^. Oxaliplatin is known from lower resistance than cisplatin and carboplatin. In human oncology, oxaliplatin is primarily used for treatment of pancreatic, gastric and colorectal cancers. To a lesser extent, it is applied during therapy of esophageal cancer. This drug is often used in some regimens as the key component: FOLFOX, FOLFIRINOX and FOLFOXIRI (FOL—folinic acid, F—5-fluorouracil, OX—oxaliplatin, IRIN/IRI—irinotecan) (Figure 1). The role of 5-fluorouracil is preventing enzymes from DNA synthesis. Folinic acid enhances the effect of 5-fluorouracil. In turn, irinotecan inhibits the key enzyme in DNA replication (topoisomerase 1). The content of each component in the regimen depends on the treatment strategy [7,21]. In veterinary oncology, oxaliplatin is used only in experimental therapies on dogs (doses up to 140 mg/m^2^) [22].

## 3. Possible Metabolic Pathways of Platinum Cytostatics

**Figure 2:**
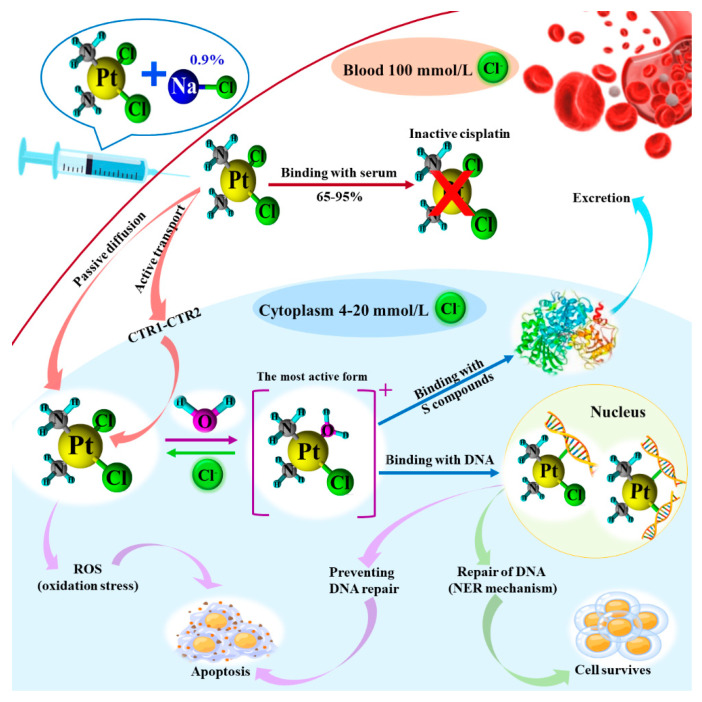
Cisplatin’s mechanism of action.

Cisplatin’s mechanism of action against cancer cells is complex (Figure 2). A saline solution of cisplatin (0.9% NaCl) is injected intravenously into the patient. In blood, cisplatin is not changed because of the relatively high Cl^−^ concentration (ca. 100 mmol/L), which stabilizes this complex platinum compound. Around 65–95% of administered cisplatin is bonded with serum proteins, which disactivate this compound. The not-bonded cisplatin flows with the blood to cancer cells. It can pass the cell membrane by passive diffusion or active transport. The latter way is directly connected to the copper transport protein (CTR1). If the CTR1 concentration is higher, then the cisplatin passing through the cancer cell membrane is more efficient. Inside the cancer cell, the Cl^−^ concentration is substantially lower (4–20 mmol/L) than in the blood. So, the cisplatin can be partially hydrolyzed with forming mono- and diaqua complexes. But the most active form of cisplatin is the monohydrolyzed one. This monoaquacisplatin (1) can be bonded with sulfur compounds (like glutathione) and excreted from the cancer cell, (2) can induce the reactive oxygen species (ROS) formation and lead to oxidation stress, and (3) can be transported to the nucleus and bonded with genomic deoxyribonucleic acid (DNA). The hydrolyzed cisplatin is bonded with DNA mainly at the N7 position of the imidazole ring of guanine. Additionally, it can be bonded with one DNA strand (monoaquated cisplatin) or with two DNA strands (diaquated cisplatin). The altered DNA can be recognized by high mobility group (HMG) proteins, nucleotide excision repair (NER) proteins or mismatch repair (MMR) proteins. There can be various scenarios of action. In the first one, the recognized error in DNA molecules can be repaired according to the NER mechanism and then the cancer cell will survive. In another scenario, HMG proteins can protect cisplatin–DNA adducts from recognition by DNA repair enzymes or modulate cell cycle events after DNA damage, which can lead to apoptosis. Another option is cell cycle arrest caused by cisplatin itself, which causes it to reenter the cell cycle prematurely in the face of unrepaired DNA damage. The damaged DNA can be replicated, which can lead to cancer cell death. Also, the oxidation stress induced by the ROS related to cisplatin can lead to apoptosis [3,7,11]. Carboplatin’s mechanism of action is similar to that of cisplatin, but the hydrolyzed products are less reactive than hydrolyzed cisplatin. Oxaliplatin’s mechanism of action is similar to that of cisplatin and carboplatin but with a few differences. This platinum drug forms up to 17 various reactive species with chlorides, methionine and glutathione, from which the main one is [Pt(DACH)(Cl)(OH_2_)]^+^ (DACH—diamminecyclohexane ligand). At an early timepoint after administration, the oxaliplatin is mainly transported to the cancer cell by the passive way. With the passage of time, oxaliplatin transport inside the cancer cell is driven by the active way with some transporters: OCT1, OCT2 and OCT3 (OCT—organic cation transporter) from the SLC22 family (SLC—solute carrier) or CTR1 and CTR2 from the SLC31A family. Partially hydrolyzed oxaliplatin bonds with DNA similarly to the cisplatin drug with the formation of suitable adducts. The oxaliplatin–DNA adducts are more effective in limiting DNA synthesis by the cancer cell. Moreover, the DNA conformation alterations induced by oxaliplatin are not detected by MMR proteins. This is an obstacle for the cancer cell to repair the damaged DNA and it leads to apoptosis. Oxaliplatin also induces immunogenic cell death by the disorder of the ribosome biogenesis. It activates nucleolar stress response pathways, which also lead to cancer cell apoptosis [7,8,9,10].

As it was mentioned earlier, the excretion of platinum-based cytostatic drugs occurs mainly with urine, which next is mixed with wastewater and goes to the environment. The proper strategy of platinum-based drug removal from the environment should be based on the knowledge of its species present in the wastewater (metabolites). Cisplatin is present in the urine of oncology patients, mainly in its intact form. Besides that, in the urine, cisplatin metabolites can also occur: predominantly monoaquacisplatin, which is a more reactive compound than cisplatin itself, and other platinum species like Pt–creatinine, cisplatin–urea and cisplatin–uric acid complexes present to a small extent [23,24]. In the case of carboplatin in patients’ urine, its intact form is presented, because this platinum-based drug is very stable [23]. In contrast, oxaliplatin is known as a very unstable drug in chloride medium. It decomposes to form the [Pt(DACH)Cl_2_] compound. Thus, it is administered by the intravenous route with 5% glucose solution to prevent its chemical changes. In the urine of patients, more than 15 various metabolites of oxaliplatin and the intact drug itself are observed. One of the detected oxaliplatin metabolites is the [Pt(DACH)Cl_2_] compound [23,24,25,26]. According to our knowledge, other oxaliplatin metabolites present in urine are not identified.

The mode of action of Pt metabolites in the environment is similar to that in living cells.

## 4. Environmental Impact of Platinum-Based Metabolites

The continuously growing number of cancer patients entails greater use of Pt-based cytostatics. The main excretion route of platinum-based metabolites is the patient’s urine, where about 75% of the administered drug is present, both in intact and metabolized hydrolyzed forms. The excretion of various Pt-based metabolites is different. During the first 4 h. of administration, about 43–48% of cisplatin and 85–91% of carboplatin is excreted. It can be related to the fact that carboplatin is bonded with plasma proteins to a smaller extent than cisplatin. The urine can easily enter the hospital and municipal wastewaters and constitute serious pollution of the aquatic environment. The concentration of Pt in hospital wastewater is between 0.01 µg/L and 762 µg/L [4,27,28,29,30,31,32,33]. In water, Pt-based cytostatics are readily soluble and undergo the hydrolysis process but in a different extent. The fastest hydrolysis occurs in the case of cisplatin [34]. On the contrary, carboplatin and oxaliplatin hydrolyze much slower than cisplatin [35,36]. The hydrolyzed forms of Pt-based drugs are readily reactive and toxic, especially monoaquacisplatin. It can strongly interact with both aquatic organisms and the abiotic matrix. It can be easily adsorbed on the sediment surface, due to the electrostatic interactions of the neutral or positively charged Pt-compounds and the negatively charged sediment particles and by surface complexation [34,35,36]. Thus, sediments can be a reservoir of Pt in the aquatic environment. These compounds can also interact with dissolved oxygen, which can transform them into Pt(IV) cytostatic compounds [37]. The presence of various inorganic/organic ligands in water also can affect the Pt speciation form. The toxicity of Pt-based metabolites on some aquatic living organisms has been studied in detail. The induction of DNA strand breaking, cell deformity, reproduction inhibition, inhibition of photosynthetic pigment synthesis, reduction in protein production or induction of oxidative stress in living cells are observed after cisplatin exposure in *Daphniamagna*, *Ceriodaphnia dubia*, *Tetrahymena pyriformis*, *Brachionus calyciflorus*, *Nereis diversicolor* and *Chlorella vulgaris*. The toxic effects of Pt-based metabolites can be observed even in ng/L concentrations. Long-term exposure to Pt-based metabolites can lead to chronic genotoxic effects in living organisms, also in animals and humans. Thus, the efficient removal of Pt-based metabolites directly from hospital wastewater or patients’ urine is of interest [30,33,38,39,40,41,42].

## 5. Removal Methods of Platinum Metabolites

Platinum-based metabolites coming from home discharge, hospital discharge and pharma industry effluents flow into conventional wastewater treatment plants (CWWTPs) [42]. CWWTP technologies are divided into three main groups: (1) primary treatment, (2) secondary treatment and (3) tertiary treatment.

In primary treatment, large particles, oil and fats (the preliminary treatment) and fine solid particles (the sedimentation/chemical precipitation) are removed from the treated wastewater. The large particles could seriously damage or clog the mechanical instruments used in CWWTPs, and oil/fats/organic solids/colloids could interfere with biological treatment methods and reduce the disinfection efficiency. The removal of organics decreases the oxygen demand of the treated wastewater. During the preliminary treatment, some physical processes are used: screening, grit or skimming. In turn, the lighter particles are removed from the wastewater by sedimentation or chemical precipitation, the latter using coagulants (e.g., alum, ferric chloride, ferric sulfate). According to R. Falter et al. [43], the relative adsorption values of cisplatin and carboplatin onto sediments present in wastewater were various (80% and 50%, respectively). The precipitation of these Pt-based compounds with iron salts was negligible (20%). Similar observations were described by the Turner research group [44], where the removal efficiency of cisplatin, carboplatin and oxaliplatin were 45%, 35% and 7%, respectively. It could be related to the weaker interactions of carboplatin and oxaliplatin with inorganic particles present in the wastewater than in the case of the cisplatin. Additionally, the coprecipitation method used in CWWTPs is ineffective for Pt-based metabolites. The hydrolysis products of cisplatin, carboplatin and oxaliplatin possess neutral or positive charge [34,35,36]. Thus, they can be partially adsorbed onto negatively charged sediments [45]. Not only ion exchange but also the complexation mechanism should be taken into account during the process of Pt-based metabolites’ adsorption onto sediments, which can contain not only inorganic but also organic matter [46]. The presence of cisplatin, carboplatin and oxaliplatin in the oil phase is negligible, due to their hydrophilic character (logK_ow_ values below zero [47]). Thus, the primary treatment methods are inefficient for Pt-based metabolite removal from wastewater, and these drugs can pass further.

The wastewater after primary treatment is subjected to secondary treatment methods. These treatment methods are dedicated to biodegradable soluble organic compounds. The main following biological treatment methods are suspended and attached growth systems. In the first type of method, free-floating microorganisms (e.g., bacteria) are suspended in aerated wastewater. The organic matter and nutrients present in the wastewater are consumed by the bacteria, which then transform them into inorganic gases and new microbial cells. Biological flocs are formed and they settle down as a sludge. The settled biomass is partially returned to the system. In the attached growth system, bacteria are retained on the media surface (gravel, ceramic, plastic) forming the biofilm. The aerated wastewater with pollutants flows over the media and bacteria degrade them. The biofilm is increasingly thicker and then it is detached off as sloughing [5]. From both of these, the suspended system is the most frequently used for the treatment of municipal wastewater [48]. The activated sludge process (ASP) and its more developed form, membrane biological reactors (MBRs), are the most applied systems for municipal wastewater treatment. In ASP technology, the effluent from the primary treatment stage passes to an aeration tank filled with a dense microbial culture in suspension (activated sludge). The ASP technology suffers from some drawbacks, like the production of excess sludge, liquid–solid separation problems, large space requirements or limitations with recalcitrant removal. In turn, MBRs are more developed than ASP, combining the biological treatment of wastewater (activated sludge or attached growth) with membrane separation (microfiltration or ultrafiltration). In MBR technology, porous polymeric membranes are often used. It prevents sediments, bacteria, small particles and degraded substances from approaching the treated wastewater. The application of MBR technology leads to the improvement of pollutant removal efficiency and selectivity, a reduction in the spatial requirements of treatment facilities, decreased cost and the generation of high-quality effluents [3,5,49,50,51]. Lenz et al. [28] studied cisplatin, carboplatin, [PtCl_4_]^2−^ and [PtCl_6_]^2−^ removal from wastewater by activated sludge in a laboratory and a pilot MBR installed in a hospital in Vienna. During laboratory tests on the activated sludge, the highest specific adsorption coefficient was estimated for cisplatin and the lowest was estimated for carboplatin. In the case of the pilot MBR studies, the total platinum elimination efficiency did not exceed 63%, which could be caused by the recalcitrant nature of the Pt-based drugs and their cytotoxicity. Additionally, the authors performed platinum species analysis of the influent and the effluent of the MBR by using HPLC-ICP-MS (high-performance liquid chromatography coupled with inductively coupled plasma mass spectrometry). It turned out that carboplatin was present in both the influent and effluent of the tested MBR as an intact drug. Platinum adsorbed onto the activated sludge of the MBR was negligible desorbed (ca. 1%) after 26 days of simulated anaerobic digestion, which suggests the irreversible adsorption of Pt compounds onto the activated sludge. The problem of the proper disposal of the platinum-loaded activated sludge should be taken into consideration. P. Verlicchi et al. [52] also suggested the high affinity of Pt-based metabolites to the activated sludge within MBRs. Moreover, the authors mentioned the application of full-scale MBRs for hospital wastewater treatment in Italy, Germany and China. New bacterial strains are developed for improving Pt-based metabolite removal from wastewater. S. R. Garakouei et al. [53] studied the removal of oxaliplatin from wastewater by *Xenorhabdus* spp., *Pantoea agglomerans* and *Bacillus licheniformis* in moving-bed biofilm reactors (MBBRs). In MBBRs, the bacterial biofilms grow on carriers and move freely in the reactor, which provides better accessibility for microorganisms to their substrate. The authors showed high oxaliplatin removal efficacy (94.0%).

After biological treatment, wastewater is subjected to tertiary treatment methods. Tertiary treatment (e.g., disinfection and reverse osmosis) is dedicated to the specific removal of biodegradable compounds containing phosphorous and nitrogen, heavy metals, viruses and pathogenic bacteria [5].

The disinfection purpose is to destroy pathogens (bacteria, viruses) using gaseous Cl_2_ or adding bleaching powder containing CaOCl_2_. In the first case, the gaseous Cl_2_ is directly purged into the wastewater with the formation of HOCl, which is responsible for destroying the pathogens, according to Equation (1):

Cl_2_(g) + H_2_O → HCl + HOCl (1)

In the second scenario, the bleaching powder is added to the wastewater and it is kept for many hours without disturbing. During this time, the following reactions occur (Equations (1) and (2)):

CaOCl_2_ + H_2_O → Ca(OH)_2_ + Cl_2_ (2)

Under disinfection conditions, platinum-based drugs present in the treated wastewater can be oxidized (change from Pt(II) to Pt(IV)) and their cytostatic properties can remain [54]. Disinfection is a low-cost and readily available method, which can be used for effective pathogen destruction. On the contrary, the used chemicals are corrosive and hazardous, and it is not a proper technique for Pt-based metabolite removal from wastewater.

Another tertiary treatment process is reverse osmosis (RO). This process is based on the application of semi-permeable membranes with tiny pores (0.5–1.5 nm) made from cellulose acetate or polyamide. As a result of osmotic pressure on both sides of the membrane (20–70 bars), only water molecules can pass through the membrane pores, and other wastewater constituents (inorganic minerals, organic compounds) are filtered [5]. The RO process is applied for the efficient removal of organic compounds, pathogens and heavy metals (also monovalent) from wastewater [55]. Thus, it could be successfully applied for cisplatin, carboplatin and oxaliplatin removal from wastewater. The process is easy to maintain and used membranes can be replaced without any problems. The disadvantages of the RO method are sludge generation, the necessity of wastewater pre-treatment, high costs related to high energy requirements and low water permeability.

CWWTPs are not sufficiently efficient for Pt-based metabolite removal from municipal wastewater, due to the recalcitrant nature of these compounds. Their concentration in the effluent can be up to 144–150 µg Pt/L [28,29]. Only in RO method could these metabolites be effectively removed from the wastewater, but it would involve high operational costs. Thus, some advanced wastewater purification technologies are studied to overcome these problems.

Advanced oxidation processes (AOPs) are initiated by strong oxidants (reactive oxygen or free radicals), which can decompose/inactivate pollutants present in the treated wastewater. Various free radicals are involved in AOPs: HO**^·^**, O_2_**^·^**^−^, HO_2_**^·^** and RO**^·^**. The most popular are HO**^·^**, due to their non-selective nature, high reactivity and powerful oxidation capabilities. These radicals react with organic compounds by the following mechanisms: hydrogen abstraction (Equation (3)), radical–radical interactions (Equation (4)), direct electron transfer (Equation (5)) and complete mineralization with production of CO_2_, H_2_O and inorganic acids [6]:

HO**^·^** + RH (R: C/N/O) → R**^·^** + H_2_O (3)

R**^·^** + O_2_ → RO_2_**^·^** (4)

HO**^·^** + RX → RX**^·^**^+^ + HO^−^ (5)

There are some processes belonging to AOPs: ozonation, ultraviolet (UV) radiation, electrolysis, photocatalysis or dielectric barrier discharge (DBD).

Hernandez et al. [36] studied cisplatin degradation in non-buffered and buffered aqueous solution by indirect reaction with ozone at pH = 9. After 2 min. of reaction, practically all initial cisplatin (>99%) was depleted. The ozone decomposed in the aqueous solution forming HO**^·^** radicals, which reacted with the cisplatin to produce mainly diamminedichlorodihydroxoplatinum(IV). The reaction proceeded with the second-order rate. Additionally, the authors observed the pH of the aqueous solutions dropping from 9.0 to 3.5 after the main product formation. The reaction mixture was not mutagenic. These studies were not performed on municipal wastewater, where not only cisplatin but also other cancerostatic Pt-based metabolites are present and the matrix is much more complicated than water. Other interactions with various compounds could occur during this process and the ozone decomposition could be performed to a lower extent, due to lower pH (ca. 7). It is related to the promotion of HO‧ formation under basic conditions [6]. Also, the cytostatic properties of this complex matrix after ozonation process could be different; thus, studies concerning municipal wastewater should be performed. The advantages of ozonation are the simple operation of this process and the absence of sludge production. On the other hand, this method suffers from high cost related to high energy demand [5,6].

The application of UV radiation for wastewater treatment is based on UV absorption by pharmaceutical molecules, the destruction of living cells initiated by UV radiation or the initiation and promotion of free radical formation by UV light. This latter strategy is dedicated to drugs with weak UV radiation absorption properties [5,6]. Lenz et al. [28] studied the application of UV light on MBR effluent containing Pt-based oncodrugs. The authors used UV radiation emitted by a low-pressure Hg lamp (energy input: 900 W/m^2^) at 254 nm for 2 min. It has been shown that 60% of carboplatin was transformed into Pt(0). It should be noticed that no change in the wastewater toxicity was observed, probably due to the presence of not-transformed carboplatin or cytotoxic carboplatin transformation products. The UV radiation method is efficient for disinfection purposes. In the case of Pt-based metabolites, the application of UV light only provides their transformation to other products, which still possess cytotoxic properties and remain in the treated wastewater.

Other AOPs include electrolysis (electrochemical oxidation), where reactive species are generated via electricity. Hirose et al. [56] studied cisplatin inactivation by the electrolysis of clinic wastewater. They used two Pt-Ir electrodes with a 5 mm gap between them. After 2 h of the electrolysis process performed under specific conditions (constant current: 100 mA and current density: 4 A/dm^2^), the cytotoxicity of the studied aqueous solution was decreased 5-fold. Studies concerning the inactivation of other Pt-based metabolites and the possible formation of electrolysis products should be performed. During the electrochemical treatment of wastewater, no additional chemicals are used. But the effectiveness of this method is strictly dependent on the electrode surface, which can be reduced by the forming products. Furthermore, the electrodes and the energy costs are high [5].

J.-F. Sauvageau et al. [57] proposed an interesting method for cisplatin inactivation from the wastewater. They used a DBD atmospheric pressure plasma reactor for that purpose. In the plasma, radical groups (mainly HO**^·^**) and solvated electrons are produced. In turn, the HO**^·^** radicals can be responsible for metal cation reduction to metal nanoparticles. The authors used Ar + 5% H_2_ plasma under specific conditions (frequency: 25 kHz, voltage: 15 kV, time: 30 min). More than 90% of cisplatin was degraded, and then it was recuperated by centrifugation as Pt-rich nanoparticles (Pt(0) and PtO, according to the XPS studies). The DBD technology would ensure the inactivation of Pt-based complexes excreted by oncology patients at the source and the efficient recovery of platinum. Additionally, the proposed method would provide a flexible Pt remediation process for hospital wastewater, because it does not require bulky installations.

Another AOP, which could be used for wastewater treatment, is photocatalysis. The most popular photocatalyst is TiO_2_ in the anatase form, due to its high photoactivity. The irradiation of TiO_2_ with UV light (300–400 nm) generates conduction of band electrons and valence band holes. Part of them recombine in the bulk of TiO_2_ material, while others reach the photocatalyst surface. The holes and electrons present on the TiO_2_ surface act as powerful oxidants and reductants, respectively. The photogenerated electrons react with the adsorbed molecular O_2_ (originating from the atmosphere or the aqueous phase) on the Ti(III) sites, reducing it to O_2_**^·^**^−^. At the same time, the photogenerated holes can oxidize directly adsorbed organic molecules or react with adsorbed OH^−^ and H_2_O producing HO**^·^** radicals. These radicals can easily attack the adsorbed organic molecules or those located close to the catalyst surface [58]. Kitsiou et al. [58] studied the photodegradation of the carboplatin (initial concentration: 20 mg/L) from an aqueous solution by TiO_2_ P25 photocatalyst with UV-A irradiation (340–400 nm). Under optimal conditions (photocatalyst concentration: 0.5 g/L, pH = 6.1, t = 60 min), 72% of carboplatin was degraded after the first 30 min of illumination. In the other 30 min, the mineralization of the organic part of this molecule to CO_2_ took place. Moreover, complete platinum deposition on the photocatalyst surface was observed. It could be related to the platinum ions’ reduction by the photogenerated electrons to Pt(0) nanoparticles. The Pt removal rate was above 80%, even after three cycles of photocatalyst usage. Additionally, a new photocatalyst was produced during the process. L. V. Barbosa et al. [59] applied a kaolinite–titanium photocatalyst for photodegradation of cisplatin from an aqueous solution. Within 90 min of the process, 67% of cisplatin was photodegraded to Pt(0), Cl^−^ and NO_3_^−^. Also, the mutagenicity of the cisplatin solution was reduced. No change in the photocatalytic activity of the studied material after six cycles was observed. Studies regarding the photocatalytic treatment of municipal wastewater, where mixed Pt-based complexes and other pharmaceuticals are present, should be performed. Also, studies should take into account realistic pharmaceutical concentrations in wastewater. The photocatalytic approach is low-cost, the process efficiency is high for the wide spectrum of pollutants and the applied photocatalyst can be easily reused, which is beneficial from the economic and environmental point of view.

The adsorption process is based on the interactions between the adsorbate ions/molecules and the porous adsorbent surface. There are many kinds of these: electrostatic interactions (attraction, repulsion or ion exchange), hydrogen bonding, hydrophobic interactions, surface complexation and surface precipitation. The interactions between adsorbent surface and adsorbate ions/molecules will affect the adsorption selectivity, the adsorption capacity and the adsorbent reusability [60]. Also, the porous structure of the adsorbent is an important factor, because it will affect the adsorption kinetics and the adsorption capacity, the latter by the specific surface area and the number of the active sites on the adsorbent surface [61]. Adsorption is the most promising technology for platinum-based drug removal from patients’ urine or hospital wastewater. This technology can be low-cost and environmentally friendly, especially when the adsorbent can be used in many adsorption/desorption cycles [62]. Moreover, this process is simple in operation and it can be used both in batch and continuous conditions. Other advantages of the adsorption method are the high removal efficiency of the pollutants even at their low concentrations and the possibility of the recovery of the adsorbed metals [63]. In the literature, there are many descriptions of the adsorbents dedicated to Pt [64,65,66,67,68,69,70,71,72,73,74,75,76,77,78,79,80,81,82,83,84,85,86,87,88,89,90,91,92,93,94,95,96,97,98,99,100,101,102,103], but there is a scarcity of information about their application for Pt-based oncological metabolites. Some of them are presented in Table 2.

K. Folens et al. [104] applied biochar, chitosan and granular activated carbon for the removal of platinum-based oncological drugs from synthetic urine. The batch adsorption conditions were as follows: C_0Pt_ = 100 µg/L, V_synth. urine_ = 20 mL, adsorbent dosage: 10 g/L, t_ads._ = 24 h. and pH_synth. urine_ = 7. The highest removal efficiencies were achieved using granular activated carbon (cisplatin: 50%, carboplatin: 60% and oxaliplatin: 95%). In the case of other studied adsorbents, the removal efficiency of Pt-based drugs did not exceed 50%. No studies regarding the adsorption process optimization (e.g., equilibrium time, adsorption capacity) were performed on the presented adsorbents for Pt-based metabolites. Also, the removal of mixed Pt complexes on the studied materials was not presented.

J. Dobrzyńska et al. [32] studied the application of a Pt(II)-imprinted thiocyanato-functionalized SBA-15 material for cisplatin, carboplatin and oxaliplatin removal from 10-fold synthetic urine. The synthesized material possessed a high specific surface area (>200 m^2^/g) and it was characterized by high selectivity towards [PtCl_4_]^2−^. The adsorption performance of the studied material was estimated only for the Pt(II) chloride complex. The Pt(II)-based metabolite removal from the diluted synthetic urine was performed in the following experimental conditions: m_adsorbent_/V_solution_ = 1 g/L, pH_0cisplatin,carboplatin_ = 1.9, pH_0oxaliplatin_ = 4.3, t_eq_ = 7 days, T = (25 ± 0.5) °C. Platinum concentrations in the diluted synthetic urine (153–206 µg/L) corresponded with that present in real hospital wastewater. The highest removal efficiency was obtained for cisplatin and oxaliplatin (>97%) and the lowest for carboplatin (ca. 94%). The authors also studied the removal of these drugs from diluted synthetic urine under pH ≈ 6 (value close to that for hospital wastewater) and they found out that the removal efficiency of cisplatin and carboplatin dropped to 76–79%. Further studies regarding the adsorption and desorption performance of the proposed material for cisplatin, carboplatin and oxaliplatin should be performed. Also, a mixed Pt-based cytostatic solution could be applied in this kind of study.

R. L. Fraguela et al. [105] synthesized a low-cost dithiocarbamate-functionalized silica gel (Si-DTC) and applied it for cisplatin removal from an aqueous solution (0.9% NaCl). The point of zero charge for the Si-DTC material was 8.6. The Si-DTC adsorption performance was studied in the cisplatin concentration range 0.5–150 mg/L, so much higher than for hospital wastewater. Under specific experimental conditions (m_adsorbent_/V_solution_ = 2 g/L, pH = 6, t_eq_ = 90 min), the maximum adsorption capacity of cisplatin on the studied material was 15.6 mg Pt/g. The authors stated that the main adsorption mechanism of cisplatin on the Si-DTC surface was surface complexation. Additionally, regeneration studies of Pt-loaded-Si-DTC were performed and the best results were obtained for 0.1 mol/L HCl (Pt removal: 60%). Selectivity studies of various cytostatic Pt-based metabolites from hospital wastewater were not performed.

T. Farias et al. [106] produced a macroporous cryogel from methacrylic acid and 2-hydroxyethyl metacrylate, which had a high affinity for cisplatin (the maximum adsorption capacity: 150 mg cisplatin/g). The equilibration of cisplatin (C_0_ = 0.5 g/L) adsorption on the studied material was reached after 24 h. The authors studied cisplatin adsorption from the aqueous solution on the synthesized material in the high concentration range (0.25–2.00 g/L), much more than for hospital wastewater. No pH influence studies were performed. Additionally, it was stated that a 2 mmol/L solution of NaOH could provide material reusability for at least 14 adsorption/desorption cycles. Studies regarding real conditions and for hospital wastewater could be performed.

The research group of D. Han [107] synthesized a cysteine-functionalized silica gel (Si-Cys) and studied its adsorption properties towards cisplatin and carboplatin from aqueous solutions. The adsorbent had a high specific surface area (480–550 m^2^/g) and a sulfur content of ca. 1 wt. %. Under the optimal experimental conditions (pH = 2, t_eq_cisplatin_ = 10 min, t_eq_carboplatin_ = 5 min, adsorbent dosage: 5 g/L) the maximum adsorption capacity of cisplatin and carboplatin was 20 mg/g. The authors stated that the main adsorption mechanism of cisplatin and carboplatin on the studied material was the surface complexation by the surface thiol groups, which provided high-affinity sites for Pt(II). Removal studies of cisplatin and carboplatin from 10-fold diluted urine at pH = 2 were performed with removal efficiency ca. 95% (cisplatin) and ca. 99% (carboplatin). Studies regarding Pt-based metabolite removal from a mixed solution under real pH conditions (pH = 6–7) could be performed. Additionally, regeneration studies of Pt-loaded adsorbents could be conducted.

F. Ogata et al. [108] applied the calcined gibbsite (specific surface area: 295 m^2^/g, pH_PZC_ = 8.1) for cisplatin removal from an aqueous solution. The maximum adsorption capacity of the cisplatin (10.6 mg/g) on the studied adsorbent was obtained under the following experimental conditions: pH = 8, t_eq_ = 24 h. and m_adsorbent_/V_solution_ = 0.4 g/L. It was shown that chemisorption was the cisplatin adsorption mechanism. Also, desorption studies were performed and the Pt was successfully removed (100%) from the Pt-loaded material by a 10 mmol/L HCl solution. There were no studies for other Pt-based metabolites present in a complex matrix, such as hospital wastewater.

D. Han et al. [109] studied a Sn/SnO_2_-coated thiol-functionalized sponge (TFS) (pH_PZC_ = 2.2, S_content_: 1.3 wt. %) for cisplatin (C_0_ = 235 µg/L) removal from an aqueous solution. The maximum adsorption capacity of the cisplatin on the studied material was 46 mg/g under the following conditions: pH_opt._ = 2–7, t_eq_ = 40 min and the adsorbent dosage: 5 g/L. The main adsorption mechanism of the cisplatin was surface complexation by thiol groups. The removal efficiency of cisplatin from an aqueous solution was >99%. The adsorption properties of the synthesized material for other Pt-based metabolites in a complex matrix (e.g., hospital wastewater) was not considered. Also, no regeneration studies of the material were performed.

According to the data collected in Table 2 for adsorbents studied for cisplatin, carboplatin and oxaliplatin removal from different matrices, it should be noted that the experimental conditions are different. It obviously hinders the comparison between various adsorbents. This is due to the significant differences in the adsorption properties of the studied materials towards cisplatin, carboplatin and oxaliplatin.

The highest affinity for Pt(II)-based oncological compounds is demonstrated by adsorbents with their surface modified by S-contained groups (e.g., thiols, dithiocarbamates or thiocyanates). This high affinity results from the chemical nature of the Pt(II) (“soft” acid) and the sulfur donors (“soft” bases) [107]. Also, other heteroatoms, like N and O, have the essential impact during Pt(II) surface complexation.

The search for proper adsorbents dedicated to the efficient Pt(II)-based metabolites from hospital wastewater or directly from patients’ urine should take into account large-scale application. The preparation cost of the materials should be as low as possible, together with the green chemistry approach. The adsorption equilibrium should be achieved in a short time period (rather minutes than hours) and the removal should be efficient at pH close to 7, corresponding to urine or hospital wastewater. Such studies should be also concerned with mixed solutions of various Pt-based oncological compounds presented at low concentrations as in WWTP influents and effluents. Moreover, the selection of the adsorption technology should consider financial aspects (e.g., Pt recovery). Not only should the adsorption process be optimized but also a Pt-loaded adsorbent treatment should be developed. Nowadays, used activated sludge with adsorbed Pt is applied in agriculture, where Pt is leached out back to the environment. One of the possible solutions is the application of regenerable carbonaceous materials for Pt-based metabolite removal from wastewater. The high number of possible regeneration cycles reduces the costs of adsorbent usage. Additionally, the used Pt-loaded carbonaceous materials could be burned to recover the precious Pt metal.

## 6. Future Perspectives

In this review, the application of platinum-based drugs in human and veterinary oncology, their mechanism of action, drug resistance, studies regarding their metabolites in patients’ urine, environmental fate and the removal technology of these compounds from hospital wastewater and directly from patients’ urine with special attention to adsorption process have been described. More studies are need to identify all of the oxaliplatin metabolites in the urine of oncological patients. Future technology for Pt metabolite removal directly from the outpatient’s urine should be based on the adsorption process, especially regarding carbonaceous materials as efficient adsorbents for this purpose. Carbonaceous materials are known as low-cost and ecofriendly materials, which can be easily chemically modified. The functionalization of carbon materials by sulfur-rich surface groups along with oxygen and nitrogen heteroatoms can enhance their affinity towards platinum compounds. Moreover, the high specific surface area of carbonaceous materials can lead to their high adsorption capacity towards platinum-based metabolites, which allows for the usage of a small adsorbent mass and decrease in costs. Also, the effective regeneration of the used materials can lead to lower technological costs. The removal of platinum-based metabolites directly from the outpatient’s urine can be the easy and ecofriendly way. Furthermore, adsorption studies should be performed in “real” conditions, regarding the concentration level of platinum-based metabolites in the patient’s urine, the urine pH, the relatively fast adsorption equilibration, the urine matrix and all platinum metabolites present in the urine. The Pt-loaded carbonaceous adsorbents can be burned and the Pt can be recovered for another usage cycle. Additionally, the proposed methods for the efficient decomposition of the Pt-based metabolites present in the patient’s urine or hospital wastewater should be further developed. Finally, application studies of the studied adsorbents at a large scale should be conducted, regarding the economic, environmental and patient safety aspects. An example could be the treatment stations present near oncological wards, to which the patient’s urine could be pumped. These systems could have the proper carbonaceous adsorbent, and after usage it could be regenerated or burned for Pt recovery. The urine of outpatients constitutes the main contribution of Pt metabolites into hospital wastewater, so such a treatment station could significantly limit their propagation to the environment. Simultaneously, efficient Pt recovery from Pt-loaded carbonaceous materials should lower the technological costs and it should provide economic profitability.

## Figures and Tables

**Table 1:** Examples of platinum cytostatic compounds used in human/animal oncology and studied by researchers.

Name /Symbol	Chemical Structure	Synthesis (Approval) Year	Clinical Use	Market Status	Ref.
cisplatin	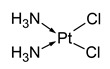	1845 (1979)	testicular, ovarian, bladder, lung, cervical, head and neck, gastric cancers, etc.	worldwide	[7,8,9,10]
carboplatin	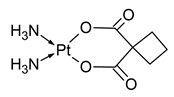	1970s (1989)	ovarian, lung, head and neck cancers, etc.	worldwide	[7,8,9,10]
oxaliplatin	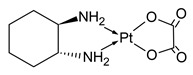	1976 (2002)	adjuvant and metastatic colorectal cancer, gastric, ovarian, pancreatic cancers, etc.	worldwide	[7,8,9,10]
picoplatin	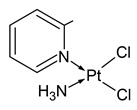	nd	ovarian, lung, hormone refractory prostate cancers, lymphoma, small intestine and colorectal cancers	clinical studies (since 1997)	[7,8,9,10]
nedaplatin	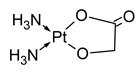	1983 (1995)	lung, esophageal, head and neck cancers, ovarian and cervical cancers, malignant urological cancers	Japan	[7,8,9,10]
lobaplatin	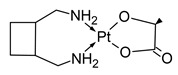	1995 (2010)	breast, lung, ovarian, cervical, intestine and stomach cancers, chronic myeloid leukemia	China	[7,8,9,10]
satraplatin	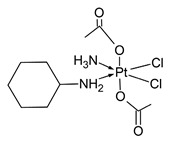	nd	prostate, lung, ovarian, cervical cancers	clinical studies	[7,8,9,10]
heptaplatin	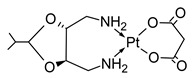	1990 (1999)	colorectal, stomach, head and neck cancers	Korea	[7,8,9,10]
pyriplatin	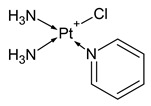	nd	nd	research studies	[7]
Glu-Pt	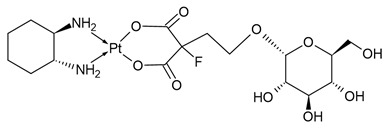	nd	nd	research studies	[7]
trans-[PtCl_2_(ipa)(dma)]	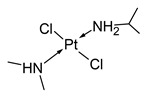	nd	nd	research studies	[7]

nd—no data; ipa—isopropylamine; dma—dimethylamine.

**Table 2:** Adsorbents used for platinum-based drugs removal from aqueous solution, hospital wastewater and urine.

Adsorbent	Matrix	Removal Conditions	Pt Removal	Adsorption Capacity	Ref.
GAC (Merck, Darmstadt, Germany)	synthetic urine	C_0Pt_ = 100 µg/L pH = 7 m/V = 10 g/L t_ads._ = 24 h.	cisplatin: 45% carboplatin: 58% oxaliplatin: 95%	nd	[104]
Chitosan	synthetic urine	C_0Pt_ = 100 µg/L pH = 7 m/V = 10 g/L t_ads._ = 24 h.	cisplatin: 36% carboplatin: 48% oxaliplatin: 40%	nd	[104]
Si-DTC	aqueous solution	C_0Pt_ = 0.5–150 mg/L pH = 6 m/V = 2 g/L t_eq_ = 90 min.	nd	15.6 mg Pt/g	[105]
Cryogel	aqueous solution	C_0cisplatin_ = 0.5 g/L m/V = 10 g/L t_eq_ = 24 h.	nd	150 mg cisplatin/g	[106]
Si-Cys	diluted urine	C_0Pt_ = 235 µg/L pH = 2 m/V = 5 g/L t_eq_cisplatin_ = 10 min. t_eq_carboplatin_ = 5 min	cisplatin: 95% carboplatin: 99%	cisplatin: 20 mg/g carboplatin: 20 mg/g	[107]
calcined gibbsite	aqueous solution	pH = 8 m/V = 0.4 g/L t_eq_ = 24 h.	nd	10.6 mg cisplatin/g	[108]
Pt(II)-imprinted thiocyanato-functionalized SBA-15	urine	C_0Pt_ = 206 µg/L (cisplatin) C_0Pt_ = 153 µg/L (carboplatin) C_0Pt_ = 174 µg/L (oxaliplatin) pH_cisplatin,carboplatin_ = 1.9 pH_oxaliplatin_ = 4.3 t = 7 days m/V = 1 g/L	cisplatin: 98.4% carboplatin: 97.1% oxaliplatin: 94.1%	nd	[33]
Sn/SnO_2_—coated TFS	aqueous solution	C_0Pt_ = 235 µg/L pH = 2–7 m/V = 5 g/L t = 40 min.	cisplatin: 99.5%	46 mg Pt/g	[109]

nd—no data; GAC—granulated activated carbon; Si-DTC—dithiocarbamate-modified silica gel; Si-Cys—cysteine-functionalized silica gel; SBA-15—Santa Barbara Amorphous No. 15; TFS—thiol-functionalized sponge.

## Data Availability

The datasets supporting the results of this article are included within the article.
